# Do differences in sport participation contribute to socioeconomic health inequalities? Evidence from the Lifelines cohort study on all-cause mortality, diabetes and obesity

**DOI:** 10.1016/j.pmedr.2023.102479

**Published:** 2023-10-31

**Authors:** Willem I.J. de Boer, Jochen O. Mierau, Ruud H. Koning

**Affiliations:** a University of Groningen, Faculty of Economics and Business, Nettelbosje 2, 9747 AE Groningen, the Netherlands; b School of Sport and Exercise, HAN University of Applied Sciences, Heyendaalseweg 141, 6525 AJ Nijmegen, the Netherlands; c Aletta Jacobs School of Public Health, Postbus 716, 9700 AS Groningen, the Netherlands

## Abstract

Little is known about the role of sport participation in socioeconomic health inequalities. We studied the association between different aspects of sport participation with all-cause mortality, type 2 diabetes mellitus (T2DM) and obesity, including inequalities between socioeconomic subpopulations. Using the Dutch Lifelines cohort study (n = 84,230), we assessed the associations of sport participation, as well as the amount, intensity, type and number of sports, with all-cause mortality, T2DM and obesity in individuals. We studied the effect of sport participation on health outcomes within and between educational categories. Outcomes were compared with moderate to vigorous physical activity (MVPA). Sport participation was significantly associated with lower mortality (HR = 0.81), T2DM (HR = 0.70), and obesity (HR = 0.77). No significant additional effects of the amount or intensity of sport participation were found, while participating in teams sport was associated with significantly lower mortality (HR = 0.53) compared with other types of sport. These effects were similar among educational categories. Sport participation explained between 11% (T2DM and obesity) and 22% (mortality) of health inequalities between educational categories. This was more than twice the effect size of MVPA. The sensitivity analysis with net income as the socioeconomic indicator showed similar results. Our results suggest that to reduce socioeconomic differences in health, public health policies should focus on increasing sport participation in groups with a low socioeconomic status, rather than increasing the amount or intensity of sport participation, or MVPA in general.

## Highlights

• Sport participation is associated with lower risks of mortality, type 2 diabetes mellitus (T2DM) and obesity.
• Doing any sports seems to be more important than the amount, type or intensity.
• Differences in sport participation contribute to 11 %–22 % of health inequalities between lower and higher educated groups.
• Health inequalities are much more explained by differences in sport participation than in physical activity in general.
• Estimates for health inequalities between low and high income groups show similar results as those for educational groups.

## 1 Introduction

Physical activity (PA) has many positive health effects, including an increased life expectancy (Andersen et al., 2000) and lower chances of being diagnosed with type 2 diabetes mellitus (T2DM) (Jeon et al., 2007). However, individuals with a lower level of education or income are likely to have higher mortality and morbidity, than those with a higher socioeconomic status (SES) (Marmot, 2015). There is very limited evidence on the specific relationship of sport participation on the socioeconomic gradient in health. The aim of this study was to investigate the association of sport participation, and aspects such as the amount and intensity, with health outcomes within as well as between socioeconomic groups. Using a large population-based panel, this is the first longitudinal study to examine the role of sport participation in the socioeconomic gradient in all-cause mortality, T2DM and obesity.

Various studies have investigated the contribution of lifestyle factors, including PA, to socioeconomic health inequalities (van Oort et al., 2004, van Lenthe et al., 2005, Silva et al., 2008, de Boer et al., 2020). A *meta*-study by Petrovic et al. (2018) demonstrated that the contribution of physical activity to socioeconomic health inequalities varied from 8 % to 17 % for mortality and from −5% to +12 % for cardiovascular diseases. However, outcomes were not completely comparable because the definition of physical activity varied between the studies (Petrovic et al., 2018). Differences in the amount, intensity, and type of physical activity may partly explain the differences across the various studies (Tanasescu et al., 2002, Sabia et al., 2012).

Sport, as a subset of PA (Khan et al., 2012), is a multidimensional concept with many different aspects, including the amount, intensity, types and number of sports practiced. Sport participation is often found to be lower among low SES groups (Kamphuis et al., 2008). In addition, sport participation is often associated with more positive health effects than other forms of PA (de Boer et al., 2021). Therefore, it is important to investigate to what extent sport participation contributes to socioeconomic health inequalities. Evidence on the relationship between the amount of participation in a specific sport and health outcomes is somewhat mixed (Oja et al., 2015).

While several studies (Oja et al., 2015, Oja et al., 2011) find a significantly positive relationship between health benefits and participating in a specific sport, Lee et al. (2016) find that the odds of coronary heart disease-related deaths were relatively increasing at higher doses of running (a reverse J-shaped association), while for cycling several studies found diminishing returns for higher volumes (Hendriksen et al., 2000, Hoevenaar-Blom et al., 2011). To our knowledge, no study has simultaneously looked at these aspects as potential drivers for the health effects of sport participation. Therefore, the research question of our study is: to what extent is sport participation itself and four different aspects (amount, intensity, types and number of sports) of sport participation associated with differences in mortality and the incidence of T2DM and obesity, between as well as within different socioeconomic groups? To put the outcomes for sport participation in perspective, we compare them with the outcomes for moderate to vigorous physical activity (MVPA) in general.

## 2 Methods

### 2.1 Sample

The Lifelines study is a multidisciplinary prospective population-based cohort study examining, in a unique three-generation design, the health and health-related behaviors of 167,729 persons living in the North of the Netherlands. It employs a broad range of investigative procedures to assess the biomedical, sociodemographic, behavioral, physical, and psychological factors that contribute to the health and disease of the general population, with a special focus on multimorbidity and complex genetics (Scholtens et al., 2015). Participants were screened through questionnaires as well as physical examination, including anthropometry. The Lifelines study was conducted according to the principles of the Declaration of Helsinki and was approved with regard to safety and privacy by the Medical Ethics Committee of the University Medical Center Groningen, The Netherlands (2007/152). Before study entry, a signed informed consent form was obtained from each participant.

Baseline measurements (1A) took place from 2006 to 2013. A full-population follow-up measurement (2A) was conducted between 2014 and 2017, with new physical examinations and questionnaires for the full (surviving) population. Intermediate questionnaire surveys (1B and 1C) were conducted with an interval of approximately 1.5 years. Mortality was registered on a monthly basis until the end of 2019. From the full cohort population, individuals with missing or implausible data for any of the variables included in our analysis were excluded. Furthermore, persons under age 25 or above 75, with T2DM, a cardiovascular disease, or cancer at baseline were excluded. For mortality, 84,230 persons remained for the analysis. Persons with incidence at baseline or an incomplete follow-up questionnaire or measurement data were excluded from the analysis about T2DM and obesity, for which the remaining sample size was 56,517 and 49,435, respectively (see flow chart Fig. A1 in the Appendix).

### 2.2 Sport participation and physical activity assessment

Sport participation and physical activity were assessed using the short questionnaire to assess health-enhancing physical activity questionnaire (SQUASH) that inquired about the frequency, intensity, duration, and type of these activities (Wendel-Vos et al., 2003). A respondent could fill out up to four sports they participated in. The amount of sport practiced was measured by the number of minutes per week. The persons who participated in sport were ordered by the amount of sport practiced and then divided into roughly equal-sized quintiles (see Table A1 of the Appendix for breakdown of the quintiles). The 2011 compendium of physical activities was used to match each physical activity type or sport discipline with a metabolic equivalent of task (MET) value as an estimate for the intensity of the activity (Weggemans et al., 2018). Total intensity was measured by summing the intensity (minutes per week multiplied with the corresponding MET-value) of all sport activities. Similar to the amount of sport, we divided total intensity in quintiles. Furthermore, we distinguished the following types of sport practiced: individual sports, semi-individual sports, team sport and fitness activities (see Table A1 of the Appendix). Finally, we investigated the number of sport as an aspect of sport participation. In our analysis, MVPA was included as a reference for the outcomes on physical activity. Physical activity with a MET-value of 4.0 or higher was categorized as MVPA, in line with the Dutch Physical Activity Guidelines for adults (Ainsworth et al., 2011). Persons performing any MVPA were divided into groups of equal size, based on the amount of MVPA in minutes per week.

### 2.3 Socioeconomic status

For SES, we distinguished three levels of education, based on the highest completed educational level of each individual: primary and practical education (low); secondary and secondary vocational education (middle) or higher education (high). For sensitivity analysis, we used net monthly household income. To obtain three roughly equal-sized groups, we categorized the income levels as follows: less than €2000; €2000-€3000; €3000 or higher (see Appendix Part B for full sensitivity analysis).

### 2.4 Outcome variables

Outcome variables in our analysis were time-to-incidence of all-cause mortality, T2DM and obesity. Mortality was registered in Lifelines on a monthly basis, and we used the data until the end of 2019. Following Deschênes et al., (2018), participants were identified as having T2DM at a follow-up period (1B, 1C or 2A) if they (1) self-reported a newly developed doctor-diagnosed T2DM; (2) were measured to have a fasting glucose value of 7.0 mmol/L or higher; or (3) had a hemoglobin A1c (HbA1c, the hemoglobin type that is bound to glucose) value of 6.5 % (Deschênes et al., 2018). Similarly, incidences of obesity occurred when respondents were measured to have a body mass index (BMI) of 30.0 kg/m^2^ or higher.

### 2.5 Analysis

For our analysis, we estimated several Cox proportional hazard regression models. The Cox model was chosen because it takes into account the time-to-event, i.e. the time between baseline measurement (1A) and the first moment of incidence; as well as time-at-risk, i.e. the time between baseline and the last measurement. In all models, sex and age were included as confounders. We performed an additional analysis that added lifestyle variables for heavy alcohol consumption, smoking, and diet quality (Dekker et al., 2017) as covariates (see Appendix Part C for the method and analysis). For the socioeconomic indicators, the highest levels (i.e. high education and high income) were taken as the reference category.

We first estimated the association of each sport participation or physical activity indicator with all-cause mortality or the incidence of T2DM and obesity. In Model 1, we included education as a covariate (see ‘Specifications of the models’ in the Appendix). Next, we examined the association of sport participation with health outcomes *within* different socioeconomic groups. Here, we estimated for each socioeconomic subpopulation a Cox proportional hazard model (Model 2). Finally, we examined the effect of sport participation on the differences *between* socioeconomic groups in the incidence of T2DM and all-cause mortality. Following the methodology of Stringhini et al. (2011), Model 3a estimates the association (hazard ratio) of individuals with low education versus those with high education (persons with middle education are not part of the model) with health outcomes. Next, this model was extended by adding each sport/physical activity variable separately (Models 3b). The contribution of each sport or physical activity indicator in explaining the association between SES and mortality (delta) was calculated as the relative reduction in the coefficient for SES after the inclusion of a physical activity indicator. For each model, we present the hazard ratios (HRs) with a 95 % confidence interval (CI). Analysis was performed using Stata 16 (Stata Corp. LLC, College Station, Texas, USA).

## 3 Results

Table 1 shows the descriptive statistics for the full sample (on mortality), stratified by education. At baseline, 56.3 % of the included persons in the sample were female and the average age was 44.5 years. Around a quarter of the sample was lower educated, while 35.4 % had completed higher education. The average follow-up time was 7.8 years for mortality (which was continuously measured until 2019), while the time between the physical measurements for T2DM and obesity was 3.4 and 3.8 years, respectively (see Table A3 in the Appendix).

**Table 1:** Summary statistics of the adult population in the Lifelines cohort study (The Netherlands) in short, by educational level (dataset mortality), at baseline (2007–2013). Proportion of the (sub-)population, unless otherwise stated.

Variable	Education			Total
	Low	Middle	High	
Number of observations	21,519	32,886	29,825	84,230
Sex (percentage females)	0.560	0.580	0.546	0.563
Average age	49.7	43.0	42.4	44.5
Education				
Low	1.000			0.255
Middle		1.000		0.390
High			1.000	0.354
Income				
Low (<€2000)	0.413	0.266	0.166	0.268
Middle (€2000-€3000)	0.394	0.399	0.266	0.351
High (>€3000)	0.194	0.334	0.568	0.381

**Sport**				
Sport participation (percentage yes)	0.451	0.565	0.695	0.582
Average mount of sport participation (min./week)	89.0	108.5	133.8	112.5
Average mount of sport participation sporters (min./week)	197.3	191.9	192.6	193.3
Average intensity of sport participation (MET-score/week)	542.5	681.2	861.0	709.4
Average intensity of sport participants (excl. no sport)	1203.0	1204.6	1239.2	1218.9
Sport types				
Individual	0.197	0.256	0.345	0.272
Semi-individual	0.086	0.102	0.160	0.118
Team	0.060	0.094	0.117	0.094
Fitness activities	0.197	0.244	0.288	0.248
Average number of sports (avg.; max 4)	0.623	0.807	1.055	0.848
Average number of sports by sport participants (avg.; max 4)	1.382	1.427	1.518	1.456

**Physical activity**				
Practicing MVPA (1 = yes)	0.812	0.847	0.896	0.856
Average amount of MVPA (min./week)	233.2	219.6	236.6	229.082

**Lifestyle**				
Smoker (1 = yes)	0.257	0.224	0.150	0.206
Alcohol heavy (>2 glasses per day)	0.119	0.099	0.127	0.114
Average Diet score	23.695	23.654	25.168	24.201
Average Body Mass Index	26.990	26.222	25.215	26.062
Overweight	0.454	0.410	0.362	0.404
Obese	0.203	0.159	0.101	0.150

**Dependent variables**				
Mortality	0.015	0.008	0.007	0.010
T2DM	0.021	0.011	0.009	0.013
Obesity	0.058	0.047	0.031	0.044

There was a clear socioeconomic gradient in sport participation, from 45.1 % of the lower educated doing sport to 69.5 % of the higher educated. Among highly educated sport participants, the amount of sport was somewhat lower, while their intensity and average number of sports was higher, compared with sport participants with low education, while also practicing more individual, semi-individual and team sports. Fitness activities were practiced more by the low educated sport participants. For MVPA, the differences between educational groups were relatively small. The incidence rate was 1.0 % for mortality, 1.3 % for T2DM, and 4.4 % for obesity. For all health outcomes, the incidence rates decreased with each step up the education ladder.

Table 2 shows the association of the indicators of sport participation and physical activity with health outcomes for the full sample (Model 1). Sport participation was significantly associated with lower mortality (HR = 0.81), less T2DM (HR = 0.70), and less obesity (HR = 0.77). The hazard ratios for participating in any sport (compared with doing no sport) were somewhat higher, but not significantly so, than the hazard ratios for doing MVPA (compared with doing no MVPA). No significant differences were found between the quintiles of either the amount or intensity of sport participation. All sport types were significantly associated with lower odds of becoming obese (HRs between 0.60 and 0.70), apart from fitness activities (HR = 0.92). Of the other three sport types, team sport had significantly lower hazard ratios for mortality (HR = 0.53) than the other sport types.

**Table 2:** Outcomes of Model 1 (full sample, with, age, sex, education as covariates) for mortality, T2DM and obesity. Hazard ratios, with 95 % confidence intervals in brackets.

Outcome	Mortality	T2DM	Obesity
Education	Full sample (all education levels)		
**Sport**			
Sport part.	0.81 (0.7–0.93)	0.7 (0.61–0.81)	0.77 (0.7–0.84)
Amount (ref = no sport)			
Q1	0.84 (0.67–1.05)	0.64 (0.5–0.82)	0.74 (0.65–0.85)
Q2	0.88 (0.71–1.09)	0.81 (0.65–1.01)	0.77 (0.68–0.88)
Q3	0.74 (0.56–0.98)	0.61 (0.45–0.83)	0.7 (0.6–0.83)
Q4	0.89 (0.7–1.13)	0.7 (0.53–0.92)	0.86 (0.74–0.99)
Q5	0.64 (0.49–0.85)	0.71 (0.54–0.93)	0.76 (0.65–0.89)
Total intensity (ref = no sport)			
Q1	0.91 (0.73–1.12)	0.75 (0.59–0.95)	0.78 (0.68–0.9)
Q2	0.8 (0.62–1.03)	0.73 (0.56–0.94)	0.73 (0.63–0.85)
Q3	0.81 (0.63–1.04)	0.7 (0.54–0.91)	0.75 (0.65–0.87)
Q4	0.8 (0.62–1.03)	0.74 (0.57–0.96)	0.77 (0.67–0.89)
Q5	0.7 (0.54–0.9)	0.59 (0.45–0.78)	0.8 (0.69–0.93)
Sport type (ref = no sport)			
Individual	0.82 (0.69–0.98)	0.59 (0.49–0.72)	0.7 (0.62–0.78)
Semi-ind.	0.76 (0.61–0.95)	0.59 (0.45–0.76)	0.6 (0.52–0.7)
Team	0.53 (0.37–0.77)	0.58 (0.41–0.8)	0.61 (0.52–0.73)
Fitness	0.88 (0.74–1.06)	0.79 (0.66–0.96)	0.92 (0.83–1.02)
Number of sports (ref = no sport)			
1	0.82 (0.7–0.96)	0.78 (0.66–0.92)	0.82 (0.74–0.9)
2	0.83 (0.67–1.02)	0.56 (0.43–0.71)	0.68 (0.59–0.78)
3	0.69 (0.46–1.06)	0.59 (0.38–0.92)	0.72 (0.57–0.92)
4 or more	0.47 (0.15–1.47)	0.58 (0.22–1.56)	0.41 (0.21–0.79)

**Physical Activity (MVPA)**			
MVPA part.	0.77 (0.64–0.93)	0.66 (0.55–0.8)	0.68 (0.61–0.77)
MVPA amount			
Q1	0.82 (0.65–1.04)	0.76 (0.6–0.96)	0.74 (0.65–0.85)
Q2	0.77 (0.6–0.99)	0.78 (0.6–1)	0.7 (0.6–0.81)
Q3	0.71 (0.55–0.91)	0.57 (0.44–0.74)	0.68 (0.59–0.79)
Q4	0.73 (0.57–0.92)	0.57 (0.44–0.74)	0.63 (0.54–0.73)
Q5	0.82 (0.66–1.02)	0.63 (0.5–0.81)	0.67 (0.58–0.78)

Note: Estimates for sport and MVPA indicators in separate regressions.

Within each educational subpopulation (Table 3), sport participation was also associated with a lower incidence (HR < 1) of all three health outcomes. For low education, sport participation was associated with significantly better odds for all health outcomes (HR = 0.78 for mortality; HR = 0.71 for T2DM; HR = 0.72 for obesity). In addition, for higher education, sport participation was associated with significantly lower mortality (HR = 0.70) and obesity (HR = 0.79), whereas for middle education, sport participation was only associated with lower odds of obesity (HR = 0.80). These associations did not differ significantly from those for MVPA, nor did they differ across SES categories. No clear health gradient in sport participation volumes or intensity was found within the SES categories. For several measures of sport participation, especially the amount and intensity, the low-education group showed a stronger gradient in reduced health risks than middle- and high-education groups. This indicates that increasing the amount or intensity of sport participation may have the greatest health benefits for the less educated. However, these differences between educational level were not significant.

**Table 3:** Outcomes of Model 2 (by education level and age and sex as covariates) for mortality, T2DM and obesity. Hazard ratios, with 95 % confidence intervals in brackets.

Outcome	Mortality			T2DM			Obesity		
Education	Low education	Middle	High	Low	Middle	High	Low	Middle	High
**Sport**									
Sport part.	0.78 (0.63–0.98)	0.89 (0.71–1.13)	0.75 (0.57–0.98)	0.71 (0.51–0.99)	0.91 (0.64–1.29)	0.78 (0.53–1.17)	0.72 (0.62–0.84)	0.8 (0.7–0.91)	0.79 (0.66–0.94)
Amount (ref = no sport)									
Q1	0.94 (0.66–1.32)	0.77 (0.51–1.16)	0.8 (0.51–1.23)	0.66 (0.38–1.15)	0.58 (0.3–1.15)	0.73 (0.38–1.39)	0.85 (0.67–1.08)	0.72 (0.59–0.9)	0.69 (0.53–0.9)
Q2	0.75 (0.51–1.1)	1.08 (0.77–1.51)	0.8 (0.53–1.2)	0.95 (0.58–1.57)	1.09 (0.66–1.78)	0.82 (0.47–1.44)	0.73 (0.56–0.94)	0.81 (0.66–0.98)	0.78 (0.61–1)
Q3	0.73 (0.45–1.19)	0.95 (0.61–1.49)	0.56 (0.32–0.97)	0.61 (0.29–1.25)	1.01 (0.54–1.89)	0.51 (0.23–1.15)	0.7 (0.51–0.96)	0.72 (0.56–0.93)	0.7 (0.52–0.93)
Q4	0.76 (0.49–1.19)	1 (0.67–1.49)	0.9 (0.59–1.38)	0.79 (0.43–1.44)	1.06 (0.6–1.87)	0.84 (0.45–1.58)	0.8 (0.61–1.05)	0.86 (0.68–1.08)	0.93 (0.72–1.2)
Q5	0.67 (0.43–1.07)	0.61 (0.37–1.01)	0.64 (0.39–1.03)	0.47 (0.22–1.02)	0.8 (0.43–1.5)	0.97 (0.54–1.76)	0.47 (0.33–0.67)	0.89 (0.7–1.13)	0.89 (0.67–1.18)
Total intensity (ref = no sport)									
Q1	1.03 (0.75–1.43)	0.8 (0.54–1.2)	0.86 (0.56–1.31)	0.85 (0.52–1.39)	0.78 (0.43–1.43)	0.69 (0.35–1.35)	0.85 (0.67–1.08)	0.75 (0.6–0.93)	0.77 (0.59–1)
Q2	0.75 (0.49–1.16)	1.1 (0.75–1.6)	0.53 (0.31–0.92)	0.77 (0.42–1.42)	0.97 (0.54–1.75)	0.98 (0.54–1.78)	0.69 (0.52–0.92)	0.79 (0.63–0.98)	0.71 (0.54–0.93)
Q3	0.51 (0.3–0.86)	1.07 (0.73–1.57)	0.86 (0.56–1.33)	0.57 (0.29–1.13)	1.08 (0.62–1.89)	0.55 (0.27–1.14)	0.7 (0.52–0.93)	0.75 (0.59–0.94)	0.81 (0.63–1.05)
Q4	0.83 (0.54–1.28)	0.74 (0.47–1.17)	0.82 (0.53–1.26)	0.75 (0.4–1.41)	0.95 (0.53–1.7)	1.11 (0.64–1.92)	0.71 (0.53–0.96)	0.76 (0.6–0.95)	0.85 (0.65–1.1)
Q5	0.67 (0.42–1.04)	0.78 (0.51–1.2)	0.64 (0.41–1.02)	0.56 (0.28–1.11)	0.79 (0.44–1.42)	0.61 (0.31–1.2)	0.61 (0.45–0.82)	0.96 (0.77–1.19)	0.8 (0.61–1.06)
Sport type (ref = no sport)									
Individual	0.84 (0.63–1.11)	0.93 (0.69–1.25)	0.76 (0.52–1.1)	0.58 (0.37–0.92)	0.8 (0.51–1.25)	0.67 (0.41–1.09)	0.68 (0.55–0.83)	0.72 (0.61–0.86)	0.69 (0.56–0.86)
Semi-ind.	0.8 (0.54–1.18)	0.72 (0.49–1.07)	0.49 (0.24–0.99)	0.63 (0.36–1.12)	0.74 (0.4–1.36)	0.63 (0.35–1.15)	0.5 (0.36–0.69)	0.62 (0.48–0.8)	0.68 (0.52–0.88)
Team	0.7 (0.41–1.21)	0.41 (0.2–0.82)	0.79 (0.58–1.07)	0.51 (0.2–1.26)	0.99 (0.53–1.83)	0.75 (0.37–1.55)	0.5 (0.34–0.74)	0.59 (0.45–0.77)	0.75 (0.55–1.01)
Fitness	0.79 (0.58–1.07)	0.94 (0.69–1.27)	0.96 (0.69–1.33)	0.74 (0.47–1.17)	1.14 (0.74–1.77)	0.98 (0.61–1.58)	0.84 (0.69–1.02)	0.98 (0.84–1.15)	0.91 (0.75–1.12)
Number of sports (ref = no sport)									
1	0.82 (0.64–1.04)	0.91 (0.7–1.18)	0.72 (0.53–0.98)	0.78 (0.54–1.12)	0.97 (0.66–1.42)	0.76 (0.48–1.18)	0.78 (0.66–0.92)	0.83 (0.72–0.96)	0.85 (0.71–1.03)
2	0.78 (0.53–1.13)	0.95 (0.66–1.35)	0.76 (0.52–1.13)	0.65 (0.38–1.13)	0.67 (0.37–1.21)	0.75 (0.43–1.3)	0.59 (0.45–0.78)	0.72 (0.58–0.88)	0.72 (0.56–0.91)
3	0.51 (0.21–1.23)	0.68 (0.32–1.45)	0.86 (0.46–1.6)	0.37 (0.09–1.51)	1.05 (0.45–2.43)	1.1 (0.52–2.36)	0.65 (0.4–1.08)	0.88 (0.62–1.25)	0.63 (0.41–0.96)
4 or more	0.47 (0.07–3.34)	n.a.	0.87 (0.21–3.51)	n.a.	1.59 (0.39–6.53)	0.86 (0.12–6.26)	0.35 (0.09–1.4)	0.24 (0.06–0.98)	0.63 (0.26–1.54)

**Physical Activity (MVPA)**									
MVPA part.	0.69 (0.53–0.9)	0.82 (0.6–1.12)	0.93 (0.61–1.41)	0.68 (0.45–1.05)	0.52 (0.34–0.79)	0.8 (0.44–1.45)	0.68 (0.57–0.83)	0.68 (0.57–0.81)	0.7 (0.55–0.89)
MVPA amount									
Q1	0.7 (0.49–1.01)	0.8 (0.53–1.2)	1.14 (0.7–1.86)	0.81 (0.47–1.37)	0.75 (0.45–1.26)	0.84 (0.41–1.72)	0.79 (0.63–1)	0.72 (0.58–0.89)	0.73 (0.55–0.98)
Q2	0.62 (0.41–0.93)	0.93 (0.61–1.41)	0.87 (0.51–1.49)	0.78 (0.44–1.4)	0.54 (0.29–0.98)	0.78 (0.37–1.67)	0.72 (0.55–0.94)	0.73 (0.58–0.92)	0.64 (0.47–0.88)
Q3	0.68 (0.47–0.99)	0.75 (0.49–1.15)	0.77 (0.45–1.31)	0.49 (0.26–0.92)	0.53 (0.3–0.95)	0.68 (0.32–1.44)	0.68 (0.52–0.87)	0.64 (0.51–0.81)	0.75 (0.56–1)
Q4	0.56 (0.38–0.83)	0.81 (0.53–1.22)	0.97 (0.59–1.59)	0.8 (0.46–1.36)	0.31 (0.16–0.62)	0.8 (0.39–1.66)	0.66 (0.51–0.85)	0.6 (0.47–0.76)	0.64 (0.47–0.87)
Q5	0.81 (0.59–1.11)	0.82 (0.55–1.22)	0.9 (0.55–1.47)	0.57 (0.33–0.98)	0.47 (0.26–0.84)	0.86 (0.43–1.74)	0.58 (0.45–0.74)	0.73 (0.58–0.91)	0.72 (0.53–0.97)

Note: Estimates for sport and MVPA indicators in separate regressions.

Results for the role of sport participation and physical activity characteristics in explaining the associations between education and mortality, T2DM, and obesity are presented in Table 4. Sport participation reduced the hazard ratio for socioeconomic inequality with 22.3 % for mortality, 11.8 % for T2DM, and 11.0 % for obesity. When the amount, total intensity, number of sports, and – to a lesser extent – sport types were included in the models (instead of sport participation), the socioeconomic hazard ratios were reduced with similar effect sizes. MVPA reduced the hazard ratio for the socioeconomic gradient with 9.0 % for mortality, 5.1 % for T2DM, and 5.3 % for obesity.

**Table 4:** Outcomes of Models 3a and 3b, showing the socioeconomic gradient in health outcomes. Hazard ratio for low education, with high education as reference.

Model	Mortality		T2DM		Obesity	
	HR^1^	Delta	HR	Delta	HR	Delta
*Model 3a (covariates: sex, age)*						
**No Sport/PA indicator**	1.254		1.564		1.677	
*Model 3b (covariates: sex, age +…)*						

**Sport**						
…+ sport participation	1.192	22.3 %	1.484	11.8 %	1.584	11.0 %
…+ sport amount	1.188	23.6 %	1.488	11.1 %	1.585	10.9 %
…+ sport intensity (total)	1.187	24.3 %	1.486	11.4 %	1.586	10.8 %
…+ sport type	1.198	20.2 %	1.462	15.1 %	1.557	14.4 %
…+ number of Sports	1.190	22.9 %	1.481	12.2 %	1.569	12.9 %
…+ amount, intensity, type & number of sports	1.183	25.6 %	1.434	19.4 %	1.532	17.6 %

**Physical activity**						
…+ MVPA participation	1.228	9.0 %	1.529	5.1 %	1.632	5.3 %
…+ MVPA amount	1.221	11.8 %	1.526	5.5 %	1.629	5.6 %

1 HR = hazard ratio for low education (with high education = 1); Delta = relative contribution of sport/physical activity participation indicator in the socioeconomic gradient, with no sport/physical activity as the reference (Model 3a).

### 3.1 Sensitivity analysis

Sensitivity analysis with income as the SES indictor instead of education (see Appendix, Part B), confirmed that sport participation explains much more of the socioeconomic gradient in health outcomes than MVPA. If anything, the size effect of sport participation was larger for income-related health inequalities (around a fourfold) than for education-related health inequalities (twofold). This implies that increasing sport participation levels among low-income groups is likely to be even more effective in lowering health differences among more affluent groups than among low educational groups.

## 4 Discussion

The objective of this study was to investigate to what extent sport participation and characteristics thereof were associated with mortality, T2DM, and obesity between and within socioeconomic groups. We showed that there was a large socioeconomic gradient in health as well as in sport participation. In contrast, for individuals who practiced sports, socioeconomic differences in the amount, total intensity, types, and number of sports practiced, were relatively small. Our research outcomes show that, within each SES group, sport participation is associated with better health outcomes. The magnitude of these health effects did not differ much across SES groups. Thus, we found no evidence of a socioeconomic gradient in the effectiveness of sport participation (or the aspects thereof) on health outcomes.

Using Cox proportional hazard regressions, we demonstrated that sport participation is significantly associated with lower mortality, T2DM, and obesity. This corresponds to the consensus in the sport and health literature (Khan et al., 2012). We found no evidence for a dose–response relationship between the amount or intensity of sport participation and health outcomes. Although several contributions found various shapes of the dose–response relationship, there does not seem to be consensus on this matter (Oja et al., 2015). The size of the health effects varied between types of sports, with team sport often more potent than other sport types, while the effect of fitness activities was significantly less strong compared with the other sport types. This suggests that fitness activities may be less beneficial for health than other types of sport, which seems to contradict the outcomes of a cross-sectional study by Schroeder et al. (2017). In addition, the health effects of fitness activities seem to be larger for low SES groups, although the differences with middle and high SES groups are not significant. Explanations for these findings may be the pluralism of fitness activities and that fitness club members are much more likely to stop (and start) their membership and activities than for instance team sport members (MacIntosh and Law, 2015). Also, residual confounding, such as different motives and barriers to participate in fitness activities for specific SES groups, may explain the differences in health effects between these groups. Gray et al. (2016) showed that neighborhood safety and limited knowledge of physical activity guidelines were important barriers for the low SES group, while for the high SES group time was an important barrier (Gray et al., 2016). Selection bias, including the initial health status (often worse among low SES individuals) and, sometimes as a result, doing fitness on a doctor’s advice, may have influenced the results.

We found that the contribution of sport participation in explaining the socioeconomic gradient of education varied between 11 % for obesity to 22 % for mortality. Remarkably, these effect sizes of sport participation were more than twice the effect size MVPA, which varied from 5 % for obesity to 9 % for mortality. This latter outcome is consistent with the reduction found for all-cause mortality in the French GAZEL study (8 %) but much less than that in the British Whitehall II study (21 %) (Stringhini et al., 2011). Our findings suggest that, more than for general PA, improving sport participation among low SES groups may be an important means to battle socioeconomic health inequalities. This is in line with the findings of de Boer et al. (2020) who showed that sport participation was more strongly related to socioeconomic inequalities in health care costs than compliance with general physical activity guidelines.

The strengths of this research are the population size of the Lifelines cohort and the longitudinal study design, including detailed assessment of physical activities using the extensive SQUASH questionnaire. We used MET values rather than self-reported energy expenditures. Although our research followed the concepts of other studies, we delved deeper into the role of sport participation, and physical activity in general, than any other study on socioeconomic differences in health.

The limitations of our research must also be noted. First, the relatively small period between baseline and follow-up measurements led to a low number of incidence. This hampered the statistical power of the outcomes, especially when stratifying for SES. Future research with more waves of the Lifelines cohort study may lead to more robust results. Second, we must take into account that PA, sport participation, and health are not independent. For instance, overweight and obesity can be the result of (previous) sport behavior, but they may also lead to reduced possibilities to be physically active or participate in a specific (type of) sports. Therefore, conclusions about causality cannot be drawn on the basis of the outcomes of this research. In particular, selection biases for the several ways of participating in sport may be based on, for example, the initial health status or health literacy. In addition, these outcomes may be (partly) a reflection of other confounding factors, such as personal preferences or environmental barriers. Third, without follow-up measurement of physical activity and sport participation, our results do not consider changes in sport and physical activity behavior. Since sport and physical activity generally declines with age, our findings may underestimate their effects on health outcomes (Gabrys et al., 2021, Eime et al., 2016). Fourth, we categorized fitness activities as a type of sports, while some scholars excluded fitness activities from sport. This limits the comparability of the findings. Finally, the external validity of this research may be somewhat limited by the specific context of Dutch society, which has a relatively flat socioeconomic gradient, a large nationwide health care system, and a large voluntary sport club infrastructure.

## 5 Conclusion

The aim of this study was to investigate to what extent sport participation and several of its aspects were associated with health outcomes and whether there were differences between as well as within different socioeconomic groups. We found that sport participation was associated with significantly lower mortality, T2DM, and obesity. We did not find evidence for additional dose–response effects in terms of the amount, total intensity, or number of sports practiced. There are some differences between types of sport, with fitness activities being associated with significantly fewer health benefits than individual, semi-individual, and, especially, team sports. Within each SES group, sport participation was positively associated to health outcomes, but we did not find evidence for differences across SES categories. However, differences in health outcomes between SES categories can (partly) be attributed to sport participation. This contribution is more than twice as large as that for MVPA. Hence, socioeconomic health inequalities seem to be mainly the result of socioeconomic differences in sport participation rather than of differences in the effectiveness of sport participation across SES groups. Our results suggest that to reduce the socioeconomic inequalities in health, public health policies should focus on increasing the participation levels in sport, rather than MVPA, for low SES groups.

## Funding sources

The Lifelines initiative was made possible by subsidy from the Dutch Ministry of Health, Welfare and Sport, the Dutch Ministry of Economic Affairs, the University Medical Center Groningen, Groningen University, and the Provinces in the North of the Netherlands (Drenthe, Friesland, Groningen).

## Patient consent for publication

Not required.

## Ethics approval

The Lifelines study was conducted according to the principles of the Declaration of Helsinki and was approved by the Medical Ethics Committee of the University Medical Center Groningen, The Netherlands (2007/152). Before study entry, a signed informed consent form was obtained from each participant.

## Data sharing statement

Researchers can apply for data and biomaterial by submitting a proposal to the Lifelines Research Office (LLscience@umcg.nl). The Lifelines website provides information on the application process (https://www.lifelines.nl).

## CRediT authorship contribution statement

**Willem I.J. de Boer:** Conceptualization, Methodology, Formal analysis, Writing – original draft, Writing – review & editing. **Jochen O. Mierau:** Conceptualization, Methodology, Supervision, Writing – review & editing. **Ruud H. Koning:** Conceptualization, Methodology, Supervision, Writing – review & editing.

## Declaration of Competing Interest

The authors declare that they have no known competing financial interests or personal relationships that could have appeared to influence the work reported in this paper.

## Data Availability

Researchers can apply for data and biomaterial by submitting a proposal to the Lifelines Research Office (LLscience@umcg.nl). For the application process see: http://www.lifelines.nl
