# Supplementary material for: Do differences in sport participation contribute to socioeconomic health inequalities? Evidence from the Lifelines cohort study on all-cause mortality, diabetes and obesity
Source: Prev Med Rep. 2023 Oct 31;36:102479. doi: 10.1016/j.pmedr.2023.102479 (PMC10641690; doi:10.1016/j.pmedr.2023.102479)
Supplement: Supplementary data 1 [file mmc1.docx]

**Supplement**

**PART A: additional information on data, models specification and outcomes**

**Figure A1**

Flow chart data selection Lifelines

**Table A1**

Classification of quintiles of the amount and intensity of sport and sport types

| Variable | Includes: |
| --- | --- |
| Amount (quintiles) | |
| Q1 | 1-60 minutes per week |
| Q2 | 61-120 minutes per week |
| Q3 | 121-180 minutes per week |
| Q4 | 181-300 minutes per week |
| Q5 | 301 minutes or more per week |
| Total intensity (quintiles) | |
| Q1 | MET-score of 1 – 420 |
| Q2 | MET-score of 421 – 684 |
| Q3 | MET-score of 685 – 1080 |
| Q4 | MET-score of 1081 – 1854 |
| Q5 | MET-score of 1855 or more |
| Amount MVPA (quintiles) | |
| Q1 | 1 – 90 minutes per week |
| Q2 | 91 – 160 minutes per week |
| Q3 | 161 – 250 minutes per week |
| Q4 | 251 – 405 minutes per week |
| Q5 | 406 minutes or more per week |
| Sport type | |
| Individual sports | Road Cycling, Mountain Biking, Running, Triathlon, (Roller) Skating, Walking, Nordic Walking, Surfing, Swimming, Ice Skating, Skiing, Snowboarding |
| Semi-individual sports | Badminton, Boxing, Fencing, Golf, Gymnastics, Horse Riding, Fight Sports (inc. Judo, Karate, Kickboxing), Billiards, Climbing, Squash, Tennis, Table Tennis, Rowing, Canoeing |
| Team sports | Basketball, Korfball, American Football, Ultimate Frisbee, Handball, Hockey, Ice Hockey, Rugby, Football, Baseball, (Beach) Volleyball, Water Polo |
| Fitness activities | Body Pump, Pilates, (Power) Yoga, Zumba, (Aqua) Spinning, Aerobics, Aquarobics, BBB, et cetera. |

**Specifications of the models**

For our analysis, we estimated several Cox proportional hazards regression models. The Cox model was chosen because it can take into account the time-to-event, i.e. the time between baseline measurement (1A) and the first moment of incidence; as well as time-at-risk, i.e. the time between baseline and the last measurement (2A for T2DM, or the end of 2019 for morbidity). In all models, sex and age were included as confounders. For the socioeconomic indicators the highest level (i.e. higher education) were taken as the reference category.

We first estimated the associations of each sport participation/physical activity indicator and all-cause mortality and the incidence of T2DM and obesity, for the full sample. In Model 1 we included education as a covariate. For each sport/physical activity indicator PAj (j = sport participation, amount of sport, total intensity of sport, sport type, number of sports, MVPA participation and MVPA amount), the hazard function of Model 1 is:

h_j_(t) = $h_{0,j}$(t) × exp ($\beta_{PA,j}$PA_j_ + $\beta_{1,j} age$+ $\beta_{2,j} sex$ + $\beta_{3,j} education$) (1)

where t represents the time between baseline measurement and the time of observation of the event (incidence of prediabetes, T2DM or mortality) or end of final observation in the case of no event. The hazard function *h_j_(t)* is determined by physical activity type j (j = sport participation, sport amount, sport intensity, sport type, number of sports, MVPA participation or MVPA amount) and a set of covariates (age, sex and education), with $h_{0,j}$(t) the hazard when covariates and sport participation are equal to zero. The quantity exp($\beta_{PA,j}$PA_j_) is the hazard ratio (HR) for each sport/physical activity type j. The hazard varies over time (t), but equals one at baseline measurement ($h$(0) = 1). A hazard ratio below one (HR <1) indicates that participating in sport leads to a smaller chance of realizing the health outcome (such as being diagnosed with prediabetes) and thus an increased length of survival. Similarly the coefficients ($\beta_{1}, \beta_{2}, \beta_{3}$) measure the effect sizes of covariates. Similar models were analyzed for the other four physical activity types.

Next, we looked at the association of sport participation on health outcomes **within** different socioeconomic groups. Here, we estimate for each socioeconomic subpopulation a Cox proportional hazard model (Model 2) with sex as age as covariates. For education K (k = low, middle or high education), Model 2 is defined as:

h_j,k_(t) = $h_{0,j,k}$(t) × exp ($\beta_{PA,j,k}$PA_j_ + $\beta_{1,j,k} age$+ $\beta_{2,j,k} sex$) (2)

with exp($\beta_{PA,j,k}$PA_j_) the hazard ratio for each physical acitivity type j in education group k.

Finally, we looked at the effect of sport participation on the differences **between** socioeconomic groups in the incidence of T2DM and all-cause mortality. Following the methodology of Stringhini et al. (2011),^26^ Model 3a estimates the association (hazard ratio) of persons with lower education (EDUlow) versus individuals with higher education (persons with middle education are not part of the model) with health outcomes:

h(t) = $h_{0}$(t) × exp (γ_0_ EDUlow + $\beta_{1} age$+ $\beta_{2} sex$) (3a)

with exp(γ_0_ EDUlow) the hazard ratio for the group with low education, with high education acting as the reference group, and covariates age and sex. Next, this model was extended by adding each sport/physical activity variable PA_j_ separately (Models 3b):

h_j_(t) = $h_{0,j}$(t) × exp (γ_j_ EDUlow + $\beta_{PA,j}$PA_j_ + $\beta_{1,j} age$+ $\beta_{2,j} sex$) (3b)

Again following Stringhini et al. (2011), we estimated the contribution of each type of PA in explaining the association between SES and mortality can be determined. For physical activity type j, this contribution (delta_j_) was calculated as the relative reduction in the coefficient for SES after inclusion of a physical activity indicator can be measured as:

delta_j_ = (ẟ_0_ - ẟ _j_) / ẟ _0_ (4)

where ẟ = log(HR) and delta_j_ represents the percent difference in the log of the hazard ratio for the socioeconomic inequality (here: low versus high education) between Model 3b or 3c (exp(γ_j_ EDUlow) and Model 3a (exp(γ_0_ EDUlow).

**Table A2**

Summary statistics, by educational level (dataset mortality)

| Variable | Education | | | Total |
| --- | --- | --- | --- | --- |
|  | Low | Middle | High |  |
| Observations | 21,519 | 32,886 | 29,825 | 84,230 |
| Sex (female = 1) | 0.560 | 0.580 | 0.546 | 0.563 |
| Age | 49.7 | 43.0 | 42.4 | 44.5 |
| Education | | | | |
| Low | 1.000 |  |  | 0.255 |
| Middle |  | 1.000 |  | 0.390 |
| High |  |  | 1.000 | 0.354 |
| Income | | | | |
| Low (<€2000) | 0.413 | 0.266 | 0.166 | 0.268 |
| Middle (€2000-€3000) | 0.394 | 0.399 | 0.266 | 0.351 |
| High (>€3000) | 0.194 | 0.334 | 0.568 | 0.381 |
| **Sport** |  |  |  |  |
| Sport participation (1=yes) | 0.451 | 0.565 | 0.695 | 0.582 |
| Amount of sport participation (min./week) | 89.0 | 108.5 | 133.8 | 112.5 |
| Amount of sport participation sporters (min./week) | 197.3 | 191.9 | 192.6 | 193.3 |
| Amount (quintiles) | | | | |
| Q0 (no sport) | 0.549 | 0.435 | 0.305 | 0.418 |
| Q1 (1-60 minutes per week) | 0.107 | 0.128 | 0.150 | 0.130 |
| Q2 (61-120 minutes per week) | 0.112 | 0.146 | 0.176 | 0.148 |
| Q3 (121-180 minutes per week) | 0.070 | 0.094 | 0.121 | 0.098 |
| Q4 (181-300 minutes per week) | 0.082 | 0.104 | 0.133 | 0.109 |
| Q5 (301 minutes or more per week) | 0.080 | 0.093 | 0.115 | 0.097 |
| Intensity of sport participation (MET-score/week) | 542.5 | 681.2 | 861.0 | 709.4 |
| Intensity of sport participants (excl. no sport) | 1203.0 | 1204.6 | 1239.2 | 1218.9 |
| Intensity (quintiles) | | | | |
| Q0 (no sport) | 0.549 | 0.435 | 0.305 | 0.418 |
| Q1 (MET-score of 1 – 420) | 0.108 | 0.118 | 0.132 | 0.121 |
| Q2 (MET-score of 421 – 684) | 0.085 | 0.112 | 0.132 | 0.112 |
| Q3 MET-score of 685 – 1080) | 0.085 | 0.114 | 0.146 | 0.118 |
| Q4 (MET-score of 1081 – 1854) | 0.083 | 0.111 | 0.143 | 0.115 |
| Q5 (MET-score of 1855 or more) | 0.090 | 0.111 | 0.142 | 0.116 |
| Average intensity (MET-score / min.) | 2.71 | 3.54 | 4.46 | 3.65 |
| Average intensity sport participation (excl. no sport) | 6.00 | 6.26 | 6.42 | 6.25 |
| Sport types | | | | |
| Individual | 0.197 | 0.256 | 0.345 | 0.272 |
| Semi-individual | 0.086 | 0.102 | 0.160 | 0.118 |
| Team | 0.060 | 0.094 | 0.117 | 0.094 |
| Fitness activities | 0.197 | 0.244 | 0.288 | 0.248 |
| Number of sport activites (by amount) | | | | |
| 0 (no sport) | 0.549 | 0.435 | 0.305 | 0.418 |
| 1 | 0.314 | 0.374 | 0.415 | 0.373 |
| 2 | 0.107 | 0.149 | 0.211 | 0.160 |
| 3 | 0.025 | 0.036 | 0.058 | 0.041 |
| 4 or more | 0.005 | 0.007 | 0.011 | 0.008 |
| Number of sport activites (avg.; max 4) | 0.623 | 0.807 | 1.055 | 0.848 |
| Number of sport activites by sporters (avg.; max 4) | 1.382 | 1.427 | 1.518 | 1.456 |
| **Physical activity** | | | | |
| Practicing MVPA (1=yes) | 0.812 | 0.847 | 0.896 | 0.856 |
| Amount MVPA (min./week) | 233.2 | 219.6 | 236.6 | 229.082 |
| Amount MVPA (quintiles) |  |  |  |  |
| Q0 (no MVPA) | 0.188 | 0.153 | 0.104 | 0.144 |
| Q1 (1 – 90 minutes per week) | 0.196 | 0.213 | 0.195 | 0.202 |
| Q2 (91 – 160 minutes per week) | 0.129 | 0.149 | 0.156 | 0.146 |
| Q3 (161 – 250 minutes per week) | 0.150 | 0.163 | 0.182 | 0.166 |
| Q4 (251 – 405 minutes per week) | 0.151 | 0.163 | 0.193 | 0.171 |
| Q5 (406 minutes or more per week) | 0.186 | 0.159 | 0.170 | 0.170 |
| **Lifestyle** | | | | |
| Smoker (1=yes) | 0.257 | 0.224 | 0.150 | 0.206 |
| Alcohol heavy (> 2 glasses per day) | 0.119 | 0.099 | 0.127 | 0.114 |
| Diet score | 23.695 | 23.654 | 25.168 | 24.201 |
| BMI (average) | 26.990 | 26.222 | 25.215 | 26.062 |
| Overweight | 0.454 | 0.410 | 0.362 | 0.404 |
| Obese | 0.203 | 0.159 | 0.101 | 0.150 |
| **Dependent variables** | | | | |
| Mortality | 0.015 | 0.008 | 0.007 | 0.010 |
| T2DM | 0.021 | 0.011 | 0.009 | 0.013 |
| Obesity | 0.058 | 0.047 | 0.031 | 0.044 |

**Table A3**

incidence of health outcomes

| Health outcome | Observations | Time at risk (years) | Avg. Follow-up time (years) | Incidence | Incidence rate (%) |
| --- | --- | --- | --- | --- | --- |
| Mortality | 84230 | 641278 | 7.6 | 822 | 0.98% |
| T2DM | 56517 | 191791 | 3.4 | 726 | 1.28% |
| Obesity | 49435 | 190101 | 3.8 | 2159 | 4.37% |

**PART B: Sensitivity analysis**

In the main text we presented the outcomes for educational levels as the measure for socioeconomic status. For additional (sensitivity) analysis we performed identical analysis with income as the measure for socioeconomic status. For income, the net monthly household income (respondents were asked to include the net income of your partner(s), if they shared a household) was assessed and combined to three roughly equal sized income categories: less than €2000; €2000-€3000; €3000 or higher. Table B1 shows the summary statistics for the datasets, stratified by income levels. Table B2 shows the outcomes (hazard ratios for sport and physical activity indicators) of the full sample model Cox proportional hazard regressions (Model 1) with income as an independent variable. In Table B3 the Model 2 outcomes for separate income levels are made visible. Finally, the outcomes of Models 3a and 3b are shown in Table B4.

**Table B1**

Summary statistics in short, by income level (dataset mortality)

| Variable | Income | | | Total |
| --- | --- | --- | --- | --- |
|  | Low (< €2000) | Middle | High (> €3000) |  |
| Observations | 22,592 | 29,541 | 32,097 | 84,230 |
| Sex (female = 1) | 0.628 | 0.548 | 0.531 | 0.563 |
| Age | 44.4 | 44.7 | 44.4 | 44.5 |
| Education | | | | |
| Low | 0.393 | 0.287 | 0.130 | 0.255 |
| Middle | 0.388 | 0.445 | 0.342 | 0.390 |
| High | 0.219 | 0.269 | 0.528 | 0.354 |
| Income | | | | |
| Low (<€2000) | 1.000 |  |  | 0.268 |
| Middle (€2000-€3000) |  | 1.000 |  | 0.351 |
| High (>€3000) |  |  | 1.000 | 0.381 |
| **Sport** | | | | |
| Sport participation (1=yes) | 0.510 | 0.548 | 0.664 | 0.582 |
| Amount of sport participation (min./week) | 102.4 | 103.0 | 128.3 | 112.5 |
| Amount of sport participation sporters (min./week) | 200.7 | 188.2 | 193.1 | 193.3 |
| Intensity of sport participation (MET-score/week) | 625.2 | 645.8 | 827.3 | 709.4 |
| Intensity of sport participants (excl. no sport) | 1225.8 | 1179.5 | 1245.1 | 1218.9 |
| Sport types | | | | |
| Individual | 0.226 | 0.252 | 0.324 | 0.272 |
| Semi-individual | 0.094 | 0.100 | 0.152 | 0.118 |
| Team | 0.077 | 0.093 | 0.105 | 0.094 |
| Fitness activities | 0.235 | 0.227 | 0.275 | 0.248 |
| Number of sports (avg.; max 4) | 0.748 | 0.774 | 0.985 | 0.848 |
| Number of sports by sporters (avg.; max 4) | 1.467 | 1.414 | 1.483 | 1.456 |
| **Physical activity** | | | | |
| Practicing MVPA (1=yes) | 0.840 | 0.852 | 0.870 | 0.856 |
| Amount MVPA (min./week) | 241.6 | 223.0 | 225.9 | 229.1 |
| **Lifestyle** | | | | |
| Smoker (1=yes) | 0.265 | 0.205 | 0.166 | 0.206 |
| Alcohol heavy (> 2 glasses per day) | 0.090 | 0.111 | 0.134 | 0.114 |
| Diet score | 23.8 | 23.9 | 24.7 | 24.2 |
| BMI (average) | 26.3 | 26.2 | 25.7 | 26.1 |
| Overweight | 0.377 | 0.416 | 0.411 | 0.404 |
| Obese | 0.180 | 0.160 | 0.119 | 0.150 |
| **Dependent variables** | | | | |
| Mortality | 0.013 | 0.010 | 0.008 | 0.010 |
| T2DM | 0.016 | 0.014 | 0.010 | 0.013 |
| Obesity | 0.051 | 0.046 | 0.037 | 0.044 |

**Table B2**

Outcomes of Model 1 (age, sex and income as covariates) for mortality, T2DM and obesity Estimates for sport and MVPA indicators in separate regressions. Hazard ratios with 95% confidence intervals.

|  | Mortality | T2DM | Obesity |
| --- | --- | --- | --- |
| **Sport** | | | |
| Sport participation | 0.82 (0.71-0.94) | 0.69 (0.6-0.8) | 0.74 (0.68-0.81) |
| Amount (reference = no sport) | | | |
| Q1 | 0.85 (0.68-1.06) | 0.63 (0.49-0.81) | 0.72 (0.63-0.82) |
| Q2 | 0.9 (0.73-1.11) | 0.8 (0.64-1) | 0.74 (0.65-0.85) |
| Q3 | 0.75 (0.57-1) | 0.6 (0.45-0.82) | 0.68 (0.58-0.8) |
| Q4 | 0.91 (0.71-1.15) | 0.69 (0.53-0.91) | 0.83 (0.72-0.96) |
| Q5 | 0.66 (0.5-0.86) | 0.7 (0.53-0.91) | 0.74 (0.63-0.87) |
| Total intensity (reference = no sport) | | | |
| Q1 | 0.91 (0.74-1.13) | 0.74 (0.58-0.93) | 0.76 (0.66-0.87) |
| Q2 | 0.82 (0.63-1.05) | 0.72 (0.56-0.93) | 0.71 (0.61-0.82) |
| Q3 | 0.82 (0.64-1.06) | 0.69 (0.53-0.89) | 0.72 (0.62-0.83) |
| Q4 | 0.81 (0.63-1.05) | 0.73 (0.56-0.94) | 0.74 (0.64-0.86) |
| Q5 | 0.71 (0.55-0.92) | 0.58 (0.44-0.77) | 0.78 (0.67-0.9) |
| Sport type (reference = no sport) | | | |
| Individual | 0.83 (0.7-0.99) | 0.58 (0.48-0.7) | 0.66 (0.59-0.74) |
| Semi-individual | 0.79 (0.63-0.99) | 0.57 (0.44-0.73) | 0.57 (0.49-0.66) |
| Team | 0.55 (0.38-0.79) | 0.56 (0.4-0.78) | 0.59 (0.5-0.7) |
| Fitness | 0.92 (0.77-1.1) | 0.77 (0.64-0.93) | 0.89 (0.8-0.99) |
| Number of sports (reference = no sport) | | | |
| 1 | 0.83 (0.71-0.97) | 0.77 (0.66-0.91) | 0.8 (0.73-0.88) |
| 2 | 0.84 (0.68-1.04) | 0.54 (0.42-0.7) | 0.65 (0.57-0.74) |
| 3 | 0.7 (0.46-1.07) | 0.57 (0.37-0.89) | 0.68 (0.54-0.87) |
| 4 or more | 0.47 (0.15-1.48) | 0.57 (0.21-1.52) | 0.39 (0.2-0.74) |
| **Physical Activity (Moderate to Vigorous Physical Activity)** | | | |
| Doing MVPA | 0.77 (0.64-0.93) | 0.64 (0.53-0.77) | 0.66 (0.59-0.74) |
| MVPA amount (reference = no MVPA) | | | |
| Q1 | 0.82 (0.65-1.04) | 0.74 (0.59-0.94) | 0.72 (0.63-0.83) |
| Q2 | 0.77 (0.6-0.99) | 0.76 (0.59-0.97) | 0.68 (0.58-0.79) |
| Q3 | 0.71 (0.56-0.91) | 0.55 (0.43-0.72) | 0.65 (0.57-0.76) |
| Q4 | 0.73 (0.57-0.93) | 0.56 (0.43-0.72) | 0.6 (0.52-0.7) |
| Q5 | 0.82 (0.66-1.02) | 0.61 (0.48-0.78) | 0.65 (0.56-0.75) |

**Table B3**

Outcomes of Model 2 (age, sex as covariates) for mortality, T2DM and obesity, by SES category (income)

| Model | Mortality | | | T2DM | | | Obesity | | |
| --- | --- | --- | --- | --- | --- | --- | --- | --- | --- |
|  | Low | Middle | High | Low | Middle | High | Low | Middle | High |
| **Sport** | | | | | | | | | |
| Sport part. | 0.65 (0.51-0.83) | 0.92 (0.74-1.16) | 0.92 (0.71-1.19) | 0.69 (0.53-0.9) | 0.75 (0.59-0.95) | 0.62 (0.47-0.8) | 0.75 (0.64-0.88) | 0.72 (0.62-0.82) | 0.75 (0.65-0.88) |
| Amount (ref = no sport) | | | | | | | | | |
| Q1 | 0.61 (0.4-0.94) | 1.03 (0.72-1.48) | 0.96 (0.64-1.45) | 0.74 (0.49-1.12) | 0.66 (0.44-1) | 0.48 (0.3-0.78) | 0.73 (0.56-0.95) | 0.73 (0.59-0.91) | 0.69 (0.54-0.88) |
| Q2 | 0.84 (0.57-1.23) | 0.93 (0.65-1.32) | 0.97 (0.67-1.42) | 0.89 (0.6-1.32) | 0.81 (0.56-1.16) | 0.7 (0.48-1.02) | 0.83 (0.65-1.05) | 0.73 (0.59-0.9) | 0.71 (0.57-0.88) |
| Q3 | 0.7 (0.42-1.17) | 0.79 (0.49-1.28) | 0.8 (0.5-1.28) | 0.68 (0.39-1.18) | 0.59 (0.35-0.99) | 0.54 (0.33-0.89) | 0.64 (0.46-0.88) | 0.68 (0.52-0.89) | 0.7 (0.54-0.9) |
| Q4 | 0.66 (0.4-1.08) | 1.06 (0.72-1.57) | 1.01 (0.67-1.52) | 0.61 (0.36-1.05) | 0.84 (0.54-1.29) | 0.6 (0.38-0.94) | 0.72 (0.54-0.97) | 0.84 (0.66-1.06) | 0.89 (0.71-1.12) |
| Q5 | 0.36 (0.18-0.71) | 0.76 (0.49-1.19) | 0.81 (0.53-1.26) | 0.4 (0.21-0.79) | 0.84 (0.55-1.28) | 0.74 (0.48-1.14) | 0.83 (0.62-1.11) | 0.58 (0.43-0.78) | 0.82 (0.63-1.05) |
| Total intensity (ref = no sport) | | | | | | | | | |
| Q1 | 0.76 (0.53-1.1) | 1 (0.7-1.43) | 1.04 (0.7-1.55) | 0.79 (0.54-1.18) | 0.75 (0.51-1.11) | 0.63 (0.4-0.99) | 0.78 (0.6-1) | 0.7 (0.56-0.88) | 0.79 (0.63-1) |
| Q2 | 0.75 (0.48-1.18) | 0.94 (0.62-1.4) | 0.8 (0.5-1.26) | 0.87 (0.56-1.38) | 0.61 (0.38-0.98) | 0.68 (0.45-1.05) | 0.78 (0.59-1.03) | 0.71 (0.56-0.91) | 0.66 (0.52-0.85) |
| Q3 | 0.54 (0.32-0.91) | 0.91 (0.6-1.37) | 1.06 (0.7-1.59) | 0.71 (0.44-1.15) | 0.77 (0.51-1.18) | 0.57 (0.36-0.91) | 0.64 (0.48-0.87) | 0.8 (0.64-1.01) | 0.69 (0.54-0.88) |
| Q4 | 0.77 (0.48-1.24) | 0.86 (0.57-1.32) | 0.85 (0.55-1.29) | 0.66 (0.39-1.13) | 0.84 (0.55-1.28) | 0.64 (0.42-0.98) | 0.8 (0.6-1.06) | 0.65 (0.5-0.84) | 0.79 (0.62-0.99) |
| Q5 | 0.34 (0.18-0.67) | 0.89 (0.59-1.33) | 0.85 (0.57-1.28) | 0.34 (0.16-0.69) | 0.77 (0.5-1.16) | 0.56 (0.36-0.88) | 0.76 (0.57-1.03) | 0.72 (0.55-0.93) | 0.83 (0.66-1.05) |
| Sport type (ref = no sport) | | | | | | | | | |
| Individual | 0.65 (0.47-0.9) | 0.92 (0.69-1.23) | 0.95 (0.69-1.3) | 0.55 (0.38-0.8) | 0.65 (0.48-0.89) | 0.51 (0.37-0.72) | 0.69 (0.56-0.85) | 0.59 (0.49-0.72) | 0.72 (0.6-0.86) |
| Semi-ind. | 0.63 (0.4-0.97) | 0.83 (0.57-1.21) | 0.89 (0.62-1.28) | 0.57 (0.35-0.94) | 0.56 (0.36-0.89) | 0.54 (0.36-0.82) | 0.53 (0.39-0.73) | 0.54 (0.41-0.7) | 0.62 (0.49-0.78) |
| Team | 0.44 (0.2-0.94) | 0.61 (0.34-1.09) | 0.59 (0.32-1.09) | 0.51 (0.25-1.05) | 0.59 (0.35-1) | 0.53 (0.31-0.9) | 0.62 (0.44-0.88) | 0.48 (0.36-0.66) | 0.68 (0.52-0.9) |
| Fitness | 0.71 (0.51-0.99) | 1.06 (0.79-1.41) | 1 (0.72-1.38) | 0.67 (0.47-0.96) | 0.92 (0.68-1.25) | 0.72 (0.52-0.99) | 0.89 (0.73-1.08) | 0.93 (0.79-1.1) | 0.86 (0.72-1.03) |
| Number of sports (ref = no sport) | | | | | | | | | |
| 1 | 0.62 (0.46-0.82) | 0.97 (0.75-1.24) | 0.94 (0.7-1.25) | 0.88 (0.66-1.16) | 0.81 (0.62-1.06) | 0.63 (0.47-0.85) | 0.81 (0.68-0.97) | 0.78 (0.67-0.9) | 0.8 (0.68-0.95) |
| 2 | 0.77 (0.52-1.13) | 0.86 (0.59-1.25) | 0.92 (0.64-1.32) | 0.4 (0.24-0.69) | 0.62 (0.41-0.93) | 0.55 (0.37-0.81) | 0.66 (0.51-0.85) | 0.62 (0.49-0.78) | 0.66 (0.53-0.82) |
| 3 | 0.61 (0.27-1.37) | 0.7 (0.33-1.5) | 0.81 (0.42-1.56) | 0.2 (0.05-0.82) | 0.52 (0.23-1.17) | 0.81 (0.46-1.45) | 0.69 (0.43-1.11) | 0.55 (0.35-0.86) | 0.78 (0.55-1.11) |
| 4 or more |  | 1.07 (0.26-4.31) | 0.46 (0.06-3.31) |  | 1.51 (0.48-4.74) | 0.33 (0.05-2.36) | 0.42 (0.13-1.3) | 0.29 (0.07-1.17) | 0.43 (0.16-1.15) |
| **Physical Activity (MVPA)** | | | | | | | | | |
| MVPA part. | 0.8 (0.58-1.08) | 0.84 (0.59-1.2) | 0.69 (0.49-0.98) | 0.77 (0.56-1.06) | 0.48 (0.35-0.66) | 0.67 (0.54-0.82) | 0.68 (0.57-0.82) | 0.62 (0.51-0.76) | 0.8 (0.58-1.08) |
| MVPA amount | | | | | | | | | |
| Q1 | 0.66 (0.44-0.98) | 0.88 (0.6-1.29) | 0.98 (0.63-1.51) | 0.81 (0.53-1.23) | 0.84 (0.57-1.25) | 0.59 (0.4-0.89) | 0.69 (0.53-0.89) | 0.8 (0.64-1) | 0.65 (0.51-0.83) |
| Q2 | 0.8 (0.53-1.21) | 0.77 (0.5-1.18) | 0.75 (0.46-1.22) | 0.86 (0.55-1.35) | 0.88 (0.57-1.35) | 0.56 (0.37-0.87) | 0.7 (0.53-0.93) | 0.78 (0.61-0.99) | 0.55 (0.42-0.71) |
| Q3 | 0.69 (0.46-1.03) | 0.73 (0.48-1.1) | 0.74 (0.46-1.18) | 0.69 (0.44-1.09) | 0.7 (0.46-1.08) | 0.31 (0.19-0.52) | 0.69 (0.53-0.91) | 0.64 (0.5-0.82) | 0.63 (0.49-0.81) |
| Q4 | 0.58 (0.38-0.89) | 0.76 (0.51-1.14) | 0.88 (0.57-1.36) | 0.61 (0.38-0.97) | 0.68 (0.44-1.04) | 0.41 (0.26-0.64) | 0.59 (0.44-0.77) | 0.61 (0.48-0.78) | 0.59 (0.46-0.76) |
| Q5 | 0.8 (0.56-1.14) | 0.82 (0.56-1.19) | 0.86 (0.56-1.32) | 0.56 (0.36-0.88) | 0.76 (0.51-1.15) | 0.53 (0.35-0.81) | 0.68 (0.52-0.88) | 0.58 (0.45-0.75) | 0.68 (0.53-0.88) |

Estimates for sport and MVPA indicators in separate regressions. Hazard ratios with 95% confidence intervals in brackets.

**Table B4**

Outcomes of Models 3a, 3b and 3c, showing the socioeconomic gradient in health outcomes (hazard ratio for low income, with high income as reference)

| Model | Mortality | | T2DM | | Obesity | |
| --- | --- | --- | --- | --- | --- | --- |
|  | HR | Delta | HR | Delta | HR | Delta |
| *Model 2a (covariates: sex, age)* | | | | | | |
| **No Sport/PA indicator** | 1.381 |  | 1.507 |  | 1.298 |  |
| *Model 2b (covariates: sex, age +…)* | | | | | | |
| **Sport** | | | | | | |
| Sport participation | 1.311 | 16.0% | 1.402 | 17.6% | 1.240 | 17.6% |
| Sport amount | 1.309 | 16.4% | 1.405 | 17.1% | 1.238 | 18.0% |
| Sport intensity (total) | 1.302 | 18.2% | 1.399 | 18.1% | 1.239 | 17.8% |
| Sport type | 1.320 | 13.8% | 1.383 | 20.9% | 1.232 | 19.9% |
| # Sports | 1.313 | 15.7% | 1.394 | 19.0% | 1.238 | 18.0% |
| Amount + intensity + type + # sports | 1.301 | 18.5% | 1.367 | 23.7% | 1.218 | 24.3% |
| **Physical activity** | | | | | | |
| MVPA participation | 1.367 | 3.0% | 1.483 | 3.9% | 1.285 | 3.9% |
| MVPA amount | 1.361 | 4.4% | 1.483 | 3.8% | 1.280 | 5.3% |
| *Model 2c (covariates: sex, age, smoking, alcohol, diet +…)* | | | | | | |
| Sport participation | 1.224 | 37.3% | 1.325 | 31.4% | 1.306 | -2.3% |
| MVPA participation | 1.260 | 28.4% | 1.417 | 15.1% | 1.359 | -17.7% |

HR = hazard ratio for low education (with high education = 1); Delta = relative contribution of sport/physical activity participation indicator in the socioeconomic gradient, with no sport/physical activity (Model 2a) as reference

**PART C: Outcomes for extended models with lifestyle factors**

In addition to the models in the main text we extended Models 2 and 3b with lifestyle factors as additional covariates. The lifestyle variables that we included were: heavy alcohol consumption (average of 2 or more glasses per day), smoking (current smoker) and diet quality (i.e. the Lifelines Diet Score),^25^ all measured at baseline. The extension of Model 2 is defined as:

h_j,k_(t) = $h_{0,j,k}$(t) × exp ($\beta_{PA,j,k}$PA_j_ + $\beta_{1,j,k} age$+ $\beta_{2,j,k} sex$ +$\beta_{3,j,k} smoking$

+ $\beta_{4,j,k} alcohol$ + $\beta_{5,j,k} diet$) (2a)

with, for example, exp($\beta_{3,j,k}$ smoking) the hazard ratio for smoking in education group k with regard to PA type j. Similarly, Model 3c adds lifestyle factors for smoking, alcohol use and diet to Model 3b:

h_j_(t) = $h_{0,j}$(t) × exp (γ_j_ EDUlow + $\beta_{PA,j}$PA_j_ + $\beta_{1,j} age$+ $\beta_{2,j} sex$

+$\beta_{3,j} smoking$ + $\beta_{4,j} alcohol$ + $\beta_{5,j} diet$) (3c)

The results of the extended models 2a are presented in Tables C1 (for educational levels) and C2 (for income). The outcomes of the extended models 3c for educational inequality are presented in Table C3, and for income inequality in Table C4.

**Table C1**

Outcomes of Model 2a for sport participation (with age, sex and lifestyle factors as covariates) for mortality, T2DM and obesity, by SES category (education)

|  | Mortality | | | T2DM | | | Obesity | | |
| --- | --- | --- | --- | --- | --- | --- | --- | --- | --- |
|  | Low | Middle | High | Low | Middle | High | Low | Middle | High |
| Sex | **0.75 (0.59-0.96)** | 0.91 (0.7-1.18) | **0.58 (0.42-0.79)** | **0.79 (0.57-1.11)** | **0.52 (0.36-0.75)** | **0.63 (0.41-0.97)** | **1.22 (1.03-1.44)** | **1.27 (1.08-1.48)** | **1.39 (1.15-1.68)** |
| Age | **1.11 (1.09-1.12)** | **1.11 (1.1-1.13)** | **1.11 (1.09-1.12)** | **1.06 (1.04-1.08)** | **1.06 (1.05-1.08)** | **1.07 (1.05-1.09)** | 1 (1-1.01) | 1 (0.99-1.01) | 1.02 (1.01-1.03) |
| Sport part. | 0.9 (0.71-1.14) | 0.96 (0.75-1.24) | 0.81 (0.61-1.08) | **0.7 (0.5-0.98)** | 0.97 (0.68-1.38) | 0.87 (0.58-1.3) | **0.67 (0.57-0.79)** | **0.78 (0.67-0.9)** | **0.79 (0.65-0.95)** |
| Smoking | **1.96 (1.49-2.57)** | **1.74 (1.3-2.33)** | **1.7 (1.19-2.43)** | 0.83 (0.52-1.33) | 1.43 (0.96-2.15) | **1.97 (1.24-3.15)** | 0.88 (0.72-1.08) | **1.25 (1.05-1.48**) | 1.14 (0.89-1.45) |
| Alcohol | **1.39 (1.05-1.83)** | 0.96 (0.69-1.33) | 1.01 (0.74-1.4) | 0.72 (0.44-1.16) | 0.99 (0.62-1.58) | 1.04 (0.65-1.65) | **0.69 (0.53-0.9)** | 0.78 (0.61-1.01) | 0.76 (0.57-1) |
| Diet | **0.96 (0.94-0.98)** | **0.97 (0.95-0.99)** | **0.96 (0.94-0.99)** | 0.99 (0.96-1.02) | 0.96 (0.93-1) | **0.95 (0.92-0.98)** | 1 (0.99-1.02) | **1.02 (1-1.03)** | **0.98 (0.97-1)** |

Estimates for sport and MVPA indicators in separate regressions. Hazard ratios with 95% confidence intervals in brackets. Significant results in **bold**.

**Table C2**

Outcomes of Model 2a for sport participation (with age, sex and lifestyle factors as covariates) for mortality, T2DM and obesity, by SES category (income)

|  | Mortality | | | T2DM | | | Obesity | | |
| --- | --- | --- | --- | --- | --- | --- | --- | --- | --- |
|  | Low | Middle | High | Low | Middle | High | Low | Middle | High |
| Sex | 0.72 (0.56-0.93) | 0.73 (0.57-0.94) | 0.73 (0.55-0.97) | 1.13 (0.85-1.5) | 0.7 (0.55-0.9) | 0.81 (0.62-1.07) | 1.44 (1.18-1.74) | 1.34 (1.14-1.57) | 1.14 (0.97-1.34) |
| Age | 1.1 (1.09-1.12) | 1.1 (1.09-1.12) | 1.11 (1.1-1.13) | 1.06 (1.04-1.07) | 1.06 (1.05-1.07) | 1.05 (1.03-1.06) | 1.01 (1.01-1.02) | 1.01 (1-1.02) | 1.01 (1-1.02) |
| Sport part. | 0.76 (0.59-0.99) | 1.05 (0.82-1.34) | 0.94 (0.72-1.23) | 0.72 (0.55-0.94) | 0.78 (0.61-0.99) | 0.64 (0.49-0.84) | 0.71 (0.59-0.84) | 0.67 (0.58-0.78) | 0.8 (0.67-0.94) |
| Smoking | 2.38 (1.8-3.14) | 1.51 (1.12-2.05) | 1.48 (1.05-2.08) | 1.1 (0.79-1.52) | 1.2 (0.88-1.64) | 0.96 (0.67-1.38) | 0.99 (0.81-1.22) | 1.17 (0.97-1.41) | 1.27 (1.04-1.55) |
| Alcohol | 1.22 (0.89-1.68) | 1.19 (0.89-1.59) | 1.05 (0.77-1.44) | 0.85 (0.56-1.27) | 0.72 (0.51-1.03) | 0.86 (0.6-1.23) | 0.59 (0.42-0.82) | 0.87 (0.68-1.1) | 0.69 (0.54-0.88) |
| Diet | 0.97 (0.95-1) | 0.96 (0.94-0.98) | 0.97 (0.95-0.99) | 0.97 (0.95-0.99) | 0.97 (0.95-1) | 0.96 (0.94-0.98) | 1 (0.98-1.01) | 1 (0.99-1.02) | 0.99 (0.97-1) |

Estimates for sport and MVPA indicators in separate regressions. Hazard ratios with 95% confidence intervals in brackets. Significant results in **bold**.

**Table C3**

Outcomes of Models 3a, 3b and 3c, showing the socioeconomic gradient in health outcomes (hazard ratio for low education, with high education as reference)

| Model (education) | Mortality | | T2DM | | Obesity | |
| --- | --- | --- | --- | --- | --- | --- |
|  | HR | Delta | HR | Delta | HR | Delta |
| ***Model 2a (covariates: sex, age)*** | | | | | | |
| No Sport/PA indicator | 1.254 |  | 1.564 |  | 1.677 |  |
| ***Model 2b (covariates: sex, age +…)*** | | | | | | |
| Sport participation | 1.192 | 22.3% | 1.484 | 11.8% | 1.584 | 11.0% |
| MVPA participation | 1.228 | 9.0% | 1.529 | 5.1% | 1.632 | 5.3% |
| ***Model 2c (covariates: sex, age, smoking, alcohol, diet +…)*** | | | | | | |
| Sport participation | 1.025 | 88.9% | 1.370 | 29.6% | 1.693 | -1.8% |
| MVPA participation | 1.050 | 78.5% | 1.425 | 20.8% | 1.786 | -12.2% |

HR = hazard ratio for low education (with high education = 1); Delta = relative contribution of PA/Sport participation indicator on the socioeconomic gradient, with no sport/PA (Model 2a) as reference.

**Table C4**

Outcomes of Models 3a, 3b and 3c, showing the socioeconomic gradient in health outcomes (hazard ratio for low income, with high income as reference)

| Model (income) | Mortality | | T2DM | | Obesity | |
| --- | --- | --- | --- | --- | --- | --- |
|  | HR | Delta | HR | Delta | HR | Delta |
| ***Model 2a (covariates: sex, age)*** | | | | | | |
| No Sport/PA indicator | 1.381 |  | 1.507 |  | 1.298 |  |
| ***Model 2b (covariates: sex, age +…)*** | | | | | | |
| Sport participation | 1.311 | 16.0% | 1.402 | 17.6% | 1.240 | 17.6% |
| MVPA participation | 1.367 | 3.0% | 1.483 | 3.9% | 1.285 | 3.9% |
| ***Model 2c (covariates: sex, age, smoking, alcohol, diet +…)*** | | | | | | |
| Sport participation | 1.224 | 37.3% | 1.325 | 31.4% | 1.306 | -2.3% |
| MVPA participation | 1.260 | 28.4% | 1.417 | 15.1% | 1.359 | -17.7% |

HR = hazard ratio for low income (with high education = 1); Delta = relative contribution of PA/Sport participation indicator in the socioeconomic gradient, with no sport/PA (Model 3a) as reference.
